# Modelling Heterogeneous Anomalous Dynamics of Radiation-Induced Double-Strand Breaks in DNA during Non-Homologous End-Joining Pathway

**DOI:** 10.3390/e26060502

**Published:** 2024-06-08

**Authors:** Nickolay Korabel, John W. Warmenhoven, Nicholas T. Henthorn, Samuel Ingram, Sergei Fedotov, Charlotte J. Heaven, Karen J. Kirkby, Michael J. Taylor, Michael J. Merchant

**Affiliations:** 1 Department of Mathematics, The University of Manchester, Manchester M13 9PL, UK; sergei.fedotov@manchester.ac.uk; 2 Division of Cancer Sciences, Faculty of Biology, Medicine and Health, The University of Manchester, Manchester M13 9PL, UK; john.warmenhoven@manchester.ac.uk (J.W.W.); nicholas.henthorn@manchester.ac.uk (N.T.H.); samuel.ingram@christie.nhs.uk (S.I.); charlotte.heaven@manchester.ac.uk (C.J.H.); karen.kirkby@manchester.ac.uk (K.J.K.); m.j.taylor@manchester.ac.uk (M.J.T.); mikejmerchant@gmail.com (M.J.M.); 3 The Christie NHS Foundation Trust, Manchester M20 4BX, UK; 4 Christie Medical Physics and Engineering, The Christie NHS Foundation Trust, Manchester M20 4BX, UK

**Keywords:** heterogeneous anomalous diffusion, chromosome aberrations, DNA damage, double-strand breaks, non-homologous end joining, dicentrics

## Abstract

The process of end-joining during nonhomologous repair of DNA double-strand breaks (DSBs) after radiation damage is considered. Experimental evidence has revealed that the dynamics of DSB ends exhibit subdiffusive motion rather than simple diffusion with rare directional movement. Traditional models often overlook the rare long-range directed motion. To address this limitation, we present a heterogeneous anomalous diffusion model consisting of subdiffusive fractional Brownian motion interchanged with short periods of long-range movement. Our model sheds light on the underlying mechanisms of heterogeneous diffusion in DSB repair and could be used to quantify the DSB dynamics on a time scale inaccessible to single particle tracking analysis. The model predicts that the long-range movement of DSB ends is responsible for the misrepair of DSBs in the form of dicentric chromosome lesions.

## 1. Introduction

Chromatin in eukaryotic cells is a highly dynamic polymer that moves within a viscoelastic medium of nucleoplasm crowded with macromolecules [1]. DNA constantly rearranges itself on large time and length scales during transcription, replication, recombination, and chromosome segregation. These processes are driven by forces generated by enzymes, molecular motors, and thermal fluctuations. DNA movement is crucial during replication and repair to maintain genome integrity and inheritance.

Chromatin mobility is a general feature of the cellular response during double-strand break (DSB) repair both at the damaged site and genome-wide [2,3,4,5]. When chromatin is damaged in the form of a DSB, the cell activates complex repair mechanisms to restore chromatin integrity. Two major repair pathways are Homologous Recombination (HR) and the canonical non-homologous end-joining pathway (c-NHEJ), which is most readily available during the cell cycle. While large-scale dynamics are required for the homology search of the sister chromatid during HR, the dynamics of DSBs during c-NHEJ are thought to be restricted and do not require extensive movement [6]. C-NHEJ involves multiple repair proteins that are recruited to the DSBs. The synapsis of DNA ends occurs in at least two stages controlled by different NHEJ factors [7]. DNA ends are initially tethered in a long-range complex whose formation requires the Ku70/80 heterodimer and the DNA-dependent protein kinase catalytic subunit (DNA-PKcs). Local remodelling allows repair proteins easier access to damaged sites.

In the absence of DNA damage, the dynamics of chromosome loci on a short time scale from seconds to minutes deviate from Brownian motion and are more accurately described by anomalous dynamics. The mean squared displacement (MSD) was found to grow sub-linearly with time, MSD $∼t^{\alpha }$, with the anomalous exponent ranging α∼ (0.3–0.5). This is in contrast with linear growth with an α=1 characteristic for Brownian diffusion [8,9,10,11,12,13,14]. Surprisingly, this behaviour is captured by a simple Rouse polymer model, which predicts α=0.5 [15].

Experiments revealed that the motility of DSB ends is also described by anomalous dynamics [5,13,16,17]. During the repair process, the distributions of displacements of DSB ends were found to be non-Gaussian, and the mean squared displacements increased sub-linearly in time, MSD $∼t^{\alpha }$ with α<1. The non-Gaussian distribution of displacements is one of the signs of the heterogeneous anomalous diffusion [18]. These experiments were described using several anomalous diffusion models. Girst et al. [16] found that a continuous time random walk (CTRW) description best fitted their experimental data, while Lucas et al. [13] reported that their experimental data showed hallmarks of Langevin and fractional Langevin motion, respectively. The polymer model predicted chromatin domain expansion near a DSB and damage extrusion from the domain after the induction of a persistent DSB [19]. Recent studies revealed the existence of directed motions during DSB repair responses (reviewed by Caridi et al. [20]). In addition to subdiffusive dynamics of chromosome loci, Pierro et al. [17] reported the existence of some spatially coherent motion of the chromatin in numerical simulations.

Chromatin motion is not purely subdiffusive even in the absence of DNA damage [21,22,23]. Several studies showed that chromatin undergoes confined random motion alternating with rare fast jumps that likely reflect rare events of active diffusion [24,25]. Levi et al. [25] thus suggested two kinds of chromatin motions in living mammalian cells: diffusive local motion and long-range movement. Heterogeneous subdiffusion with coherent motion on a micron scale was observed in interphase human chromosomes [26]. Polymer models also suggest highly heterogeneous dynamics with a broad distribution of the diffusion exponents of the individual loci [27].

Recent studies of chromatin mobility have revealed the co-existence of subdiffusion with rare long-range directed motion in living cells in response to DSBs [20,28,29]. The directed chromatin movements during DSB repair are ATP dependent [30] and appear also during RNA transcription [31]. The possible mechanism of this long-range directed motion was attributed to F-actin and regulatory mechanisms coordinating chromatin dynamics [20], nuclear microtubule filaments [29], or clustering of DSBs at damaged genes [32,33].

Traditional approaches to model the repair of DSBs either do not consider the dynamics of DSB ends or use Brownian diffusion for this purpose [34]. Since Brownian dynamics is Markovian and memoryless, this allows us to utilize the analytical expression for a Gaussian propagator and very efficient Monte Carlo simulation schemes. However, instead of Brownian diffusion, DSB motility must be modelled by anomalous dynamics. The DNA polymer models capture the anomalous dynamics of DSBs well; however, they are prohibitively computationally expensive and cannot reach the desired time scale of hours over which the repair takes place [17,19]. Therefore, recently, we have developed a mechanistic DSB repair model with the c-NHEJ pathway and DSB ends driven by the subdiffusive continuous time random walk (CTRW) model. This has been incorporated into the DNA Mechanistic Repair Simulator (DaMaRiS) [35]. This model implements subdiffusive dynamics of DSBs and can easily simulate the repair process over many hours. However, it did not include the long-range directed movement and was unable to predict any dicentric misrepair formed through the fusion of two chromosome segments, each with a centromere, since the DNA ends on different chromosomes were moving too slowly to approach each other.

In this paper, we develop a new model with heterogeneous anomalous dynamics of DSBs, which consists of subdiffusive motion modelling the DNA polymer chain dynamics and rare long-range directional movement of DSB ends. The MSD analysis is insufficient to study mixed subdiffusive and directed motion of DSBs for several reasons. First, the current technology is still limited in measuring trajectories of several minutes’ duration instead of hours over which the repair takes place. Second, the heterogeneous dynamics, which consist of switching between subdiffusive movement and rare directed motion, would be masked in ensemble-averaged MSDs [6]. Therefore, we propose to use the time behaviour of the survival function of DSBs (the number of residual DSBs divided by the initial number of DSBs) and the dose–response curve of the yield of dicentric aberrations as measures to quantify the amount of directed motion during the long period of repair inaccessible to the MSD analysis. Our results show that the rare long-range directional movement of DSB ends leads to incorrect repair in the form of dicentric aberrations.

## 2. Materials and Methods

We follow our previous work [35] in the implementation of the NHEJ process via the scheme for the time evolution of individual DSB ends. They were governed by a series of time-constant-based state changes corresponding to the recruitment of repair proteins (Ku70/80), DNA-PKcs, and the formation of synaptic complexes. We have used the same rate constants as in [35]. However, in addition to the continuous time random walk (CTRW) process that was used in [35] to model the movement of individual DSB ends, here we consider the fractional Brownian motion (FBM). The basic properties of FBM are defined below.

Dicentrics are chromosomes with two centromeres. These abnormal chromosomes result from improper DNA repair processes, particularly after cells are exposed to ionizing radiation or other agents that cause DSBs. In a normal chromosome, the centromere is crucial for proper segregation during cell division. However, when a chromosome has two centromeres (dicentric), it can lead to genomic instability, as the dicentric chromosome may get pulled in opposite directions during mitosis, potentially resulting in chromosome breakage or mis-segregation.

In our model, dicentrics are formed through the misrepair of DSBs in DNA. The cell attempts to repair these breaks through NHEJ. During this repair process, ends from different chromosomes or distant regions of the same chromosome may be incorrectly joined together. If two broken chromosome ends, each containing a centromere, are brought close to each other via one of the heterogeneous diffusion processes described below and misjoined, a dicentric chromosome is formed.

Our simulations aim to predict the formation of dicentrics based on theoretical models and various parameters (e.g., radiation dose, type of radiation, cell type) by using computational models to simulate the occurrence of DSBs and their repair. We can provide estimates of the number of dicentrics formed under specific conditions as functions of time after irradiation. This number can be compared with the empirically measured number of dicentrics formed in cells exposed to radiation, obtained using techniques such as cytogenetic analysis (e.g., fluorescence in situ hybridization, or FISH) to count dicentrics in irradiated cells. Results of these ongoing experiments and comparisons with the model predictions will be published in the future.

### 2.1. Fractional Brownian Motion (FBM)

The equations of motion are given by the overdamped Langevin equation driven by an external fractional Gaussian noise (*fGn*) ${\vec{\xi }}_{fGn}\left(t\right)$ characterized by the anomalous exponent α and the noise intensity ${\tilde{K}}_{\alpha }$, as follows:

(1) $$\frac{d\vec{r}}{dt}={\vec{\xi }}_{fGn}\left(t\right),$$

where $\vec{r}=\left\{x,y,z\right\}$ and ${\vec{\xi }}_{fGn}=\left\{\xi_{fGn,x},\xi_{fGn,y},\xi_{fGn,z}\right\}$. The noise components are independent and power law correlated, as follows:

(2) $$\langle \xi_{fGn,i}\left(t\right)\xi_{fGn,i}\left(t^{\prime }\right))\rangle ∼K_{\alpha }{\left|t-t^{\prime }\right|}^{\alpha -2}.$$

For α<1, *fGn* is anti-persistent, which leads to subdiffusion with the EMSD and TMSD growing sub-linearly with time, as follows:

(3) $$\langle {\vec{r}}^{2}\left(t\right)\rangle =\bar{\delta^{2}\left(t\right)}=6K_{\alpha }t^{\alpha }.$$

In contrast with CTRW, fBm is ergodic.

### 2.2. Simulation of Heterogeneous Movement of DSB Ends

To model the heterogeneous dynamics of DSB ends, incorporating both subdiffusion and long-range directional movement, we employ a set of stochastic Langevin equations, as follows:

(4) $$d\vec{r}\left(t\right)/dt=\vec{dr}+I\left(t\right)\vec{v}\left(t\right),$$

where $\vec{dr}$ are increments of FBM. For FBM, $\vec{dr}={\vec{\xi }}_{fGn}\left(t\right)$. The indicator function I(t)={0,1} is the two-state Markov process with transition rates $\mu_{0}=0.001$/s and $\mu_{1}=0.016$/s, which adds an active state with a constant magnitude velocity vector *v*. The result models are thus called Heterogeneous FBM (HFBM). The average duration of the active phase was approximately $1/\mu_{1}∼60$ s. The direction of the velocity was uniformly chosen from (0,2π). The magnitude of the active state was chosen as v=16 nm/s such that the average distance travelled during the active phase was 1 micron. The direction v changes randomly (using uniform distribution from (0,2π)) every active phase I(t)=1. FBMs were generated using the Fast Fourier Transform (FFT) for speed [36] with the constant anomalous exponent α=0.5 and generalized diffusion coefficient *D*. We note that the exact methods to generate *fGn* such as ffgn function from Matlab R2020b (Natick, Massachusetts) were prohibitively memory expensive here. The initial number and position of DSBs were calculated using TOPAS-nBio Monte Carlo software (TOPAS-nBio v2.0) [37]. Simulations were repeated 200 times.

## 3. Results

We have studied the heterogeneous motion of DSB ends consisting of subdiffusive motion interspersed with short periods of active transport. The CTRW model of DSB repair was implemented in our previous work [35]. However, we showed that a CTRW interpretation of the subdiffusive motion of DSB ends was likely insufficient to explain overall repair kinetics. In this work, we consider FBM instead of CTRW. One important difference between CTRW and FBM is that CTRW is non-ergodic, while FBM is ergodic in the sense of the equivalence between the ensemble and time mean squared displacement.

Mathematically, the repair of DSB ends can be considered as a diffusion–annihilation process, A+A→0. Thus, the residual DSBs are given by the survival function S(t). For Brownian diffusion–annihilation reaction in three dimensions, the long-time behaviour of the survival function decays as S(t)∼1/t [38]. For one-dimensional subdiffusive CTRW with annihilation, the long-time behaviour of the survival function was calculated as $S\left(t\right)∼t^{\alpha /2}$ [39]. Spatial limitations of the diffusion mobility of DSB ends inside bounded regions significantly change the kinetics of reaction, compared with the unbounded medium. The presence of boundaries, such as topologically associating domains (TADs), may increase the probability of DSB ends approaching the distance at which they can interact. On the other hand, the directed movement of DSB ends drives them apart and increases this distance. To our knowledge, the dependence of S(t) for subdiffusion–annihilation in three dimensions such as FBM or heterogeneous mixtures of FBM model with directed movement that we are considering here is not known. Therefore, we use numerical simulations.

**Figure 1:**
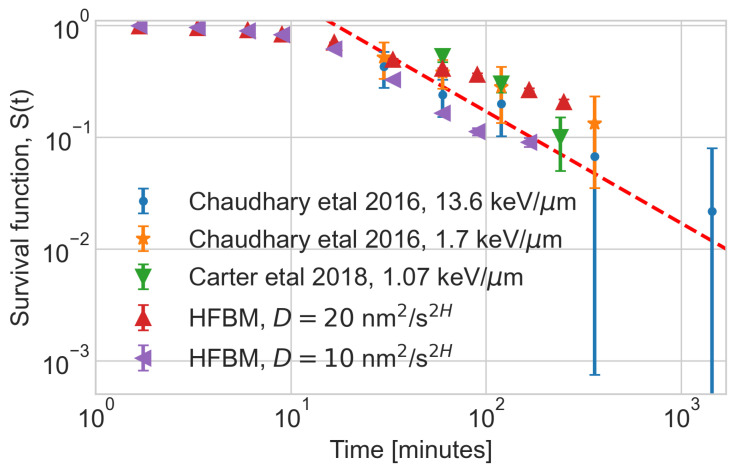
Survival function for the HFBM model compared with the experimental data for human skin fibroblast (AG01522) [40] and HeLa cell [37,41] for 20 MeV protons (2.06 keV/μm) with a 0.3 Gy dose. The dashed line represents the power law $t^{-1}$ to guide the eyes.

A bespoke application [35] utilising the Monte Carlo radiation transport framework, Geant, was used to predict the initial number and position of DSBs due to irradiation by 26 MeV protons. Although the spread of the available experimental data is large, by varying the generalized diffusion coefficient and on–off rates, we find parameters such that the survival function (defined as the number of residual DSBs divided by the initial number of DSBs) as a function of time fits the experimental data. The numerically calculated survival function for the heterogeneous FBM model (HFBM) is compared in Figure 1 with the experimental data from a work by Chaudhary et al. [40] (obtained using human skin fibroblast cells irradiated with 60 MeV therapeutic proton beam with a 1 Gy dose and two linear energy transfers, 13.6 keV/μm and 1.7 keV/μm), and from Carter et al. [41] (obtained using HeLa cells irradiated with 58 MeV protons with a 4 Gy dose). The parameters of HFBM were H=0.25, D=20 nm^2^/s^0.5^, $\gamma_{on}=0.001$ s, and $\gamma_{off}=0.016$ s. The decay of the survival function follows the inverse power law similar to the Brownian reaction–annihilation reaction. The higher value of the diffusion coefficient increases the separation of initial partner DSB ends, making the decay of the survival function slower.

**Figure 2:**
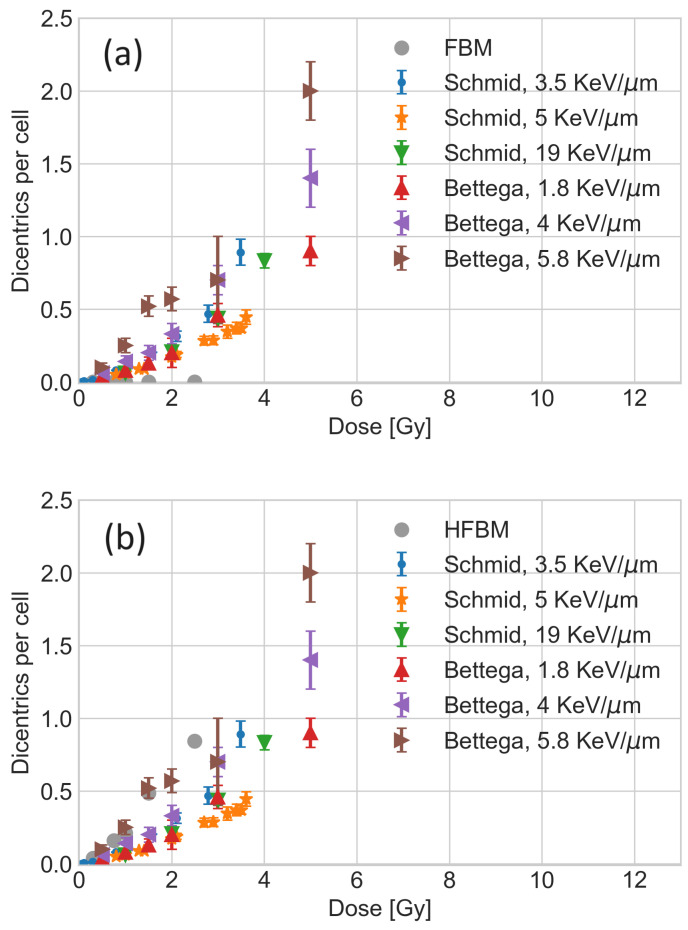
Number of dicentrics per cell after 24 h of repair as a function of the dose of proton irradiation for FBM without directed movement (**a**) and HFBM (**b**). HFBM reproduces a trend similar to experimental data from Bettega et al. [42] (blood samples) and Schmid et al. [43] (human heteroploid line with epithelioid morphology).

Long-range directed movement in a heterogeneous anomalous diffusion model drives DSB ends apart, potentially leading to severe lesions when two different chromosome segments with centromeres connect and form a dicentric segment. As dicentric chromosome aberrations almost always result in mitotic catastrophes, dicentrics can be used as a surrogate for predicted cell survival. To assess the number of dicentric mis-repairs predicted by the heterogeneous anomalous diffusion model, we calculated the dicentric dose–response curve (the number of dicentrics per cell) using DNA damage at different irradiation doses. The results are shown in Figure 2a,b. The FBM model without directed movement of DSB ends (Figure 2a) clearly shows that subdiffusion alone is not sufficient to bring DSB ends from different chromosomes close to each other and form a dicentric lesion. On the contrary, heterogeneous FBM with directed movement leads to the formation of dicentrics and reproduces the experimental dose–response trend, as shown in Figure 2b.

## 4. Discussion

Chromatin dynamics in eukaryotic cells are a highly intricate process crucial for various cellular functions such as transcription, replication, recombination, and DNA repair. In the absence of DNA damage, chromatin exhibits complex behaviours that deviate from simple Brownian motion.

How eukaryotic DNA repairs itself after radiation damage remains poorly understood today. The amount of chromosome movement and how it changes upon the induction of DSBs has been a subject of intensive investigations and remains greatly debated in the literature. It depends on how DSBs were inducted, the cell type, the cell cycle stage, and the accumulation of repair complexes. During DSB repair, the dynamics of chromatin and DSB ends are governed by anomalous diffusion, characterized by non-Gaussian distribution and sub-linear MSD growth with time [5,13,16,17]. Various models have been proposed to describe these dynamics, including continuous time random walk (CTRW) models [16] and polymer models [27]. However, traditional models often overlook the rare long-range directed motion observed during repair processes [20,28,29].

To address this limitation, we present a novel model that incorporates heterogeneous anomalous dynamics of DSBs, encompassing both subdiffusive motion of the DNA polymer chain and rare long-range directed movement of DSB ends. Unlike traditional approaches that rely on MSD analysis, our model captures the intricate interplay between subdiffusion and directed motion. This is critical as ensemble-averaged MSDs may mask the presence of rare directed movements, especially when trajectories cannot be measured over the time scales relevant for repair processes [6].

There are multiple mechanisms of long-range movement, which include the activity of nuclear microtubules, nuclear actin filaments [20], random forces produced by enzymes, and processive motors such as polymerases and topoisomerase [9]. It is likely that our heterogeneous anomalous diffusion model overestimates the amount of directed movement due to the unrealistic way of the long-range movement (e.g., random direction of each directed segment). In experiments, radial movements towards the nuclear periphery were often observed [20]. It was also suggested that DSBs could be moved to repair centres [32], which also include the possibility of dicentric lesions. Heterogeneous FBM is a non-Markovian model that makes it difficult to implement into DaMaRiS v2020.07.23 or other Monte Carlo software. We are planning to conduct this together with a more realistic modelling of long-range dynamics in our future work.

While subdiffusion supports the joining of correct ends during DNA repair by making DSB ends explore the space more recursively [16], the inter-chromosome DNA lesions such as dicentric chromosomes require DSBs on different chromosomes to move close to each other. Thus, DSB ends move heterogeneously with local subdiffusion motion and rare long-range movement. The amount of dicentrics could serve as a measure of long-range dynamics. Experimental data resulting from particle radiation are lacking in this area to be used for a more detailed comparison. The datasets used in this analysis, originating from HeLa cells, are not ideal for a comparison with the model data due to the aneuploidy of HeLa and its radioresistant nature. This lack of experimental validation datasets remains a limitation of this type of modelling. Our work presents a mechanistic approach to evaluate heterogeneous DSB dynamics computationally by juxtaposing the long-term decay of the survival function of unrepaired DSB ends and the dose–response curve of dicentric chromosomal lesions with experimental data. The model offers a more quantitative depiction of the repair process over extended time scales, which are beyond the reach of single-track imaging techniques.

## Data Availability

The data that support the findings of this study are available from the corresponding author upon reasonable request.

## References

[1] Alberts B., Johnson A., Lewis J., Raff M., Roberts K., Walter P. (2007). Molecular Biology of the Cell.

[2] Herbert S., Brion A., Arbona J.M., Lelek M., Veillet A., Lelandais B., Parmar J., Fernández F.G., Almayrac E., Khalil Y. (2017). Chromatin stiffening underlies enhanced locus mobility after DNA damage in budding yeast. Embo J..

[3] Hauer M.H., Seeber A., Singh V., Thierry R., Sack R., Amitai A., Kryzhanovska M., Eglinger J., Holcman D., Owen-Hughes T. (2017). Histone degradation in response to DNA damage enhances chromatin dynamics and recombination rates. Nat. Struct. Mol. Biol..

[4] Lawrimore J., Barry T.M., Barry R.M., York A.C., Friedman B., Cook D.M., Akialis K., Tyler J., Vasquez P., Yeh E. (2017). Microtubule dynamics drive enhanced chromatin motion and mobilize telomeres in response to DNA damage. Mol. Biol. Cell.

[5] Miné-Hattab J., Recamier V., Izeddin I., Rothstein R., Darzacq X. (2017). Multi-scale tracking reveals scale-dependent chromatin dynamics after DNA damage. Mol. Biol. Cell.

[6] Miné-Hattab J., Chiolo I. (2020). Complex chromatin motions for DNA repair. Front. Genet..

[7] Graham T.G., Walter J.C., Loparo J.J. (2016). Two-stage synapsis of DNA ends during non-homologous end joining. Mol. Cell.

[8] Bronstein I., Israel Y., Kepten E., Mai S., Shav-Tal Y., Barkai E., Garini Y. (2009). Transient anomalous diffusion of telomeres in the nucleus of mammalian cells. Phys. Rev. Lett..

[9] Weber S.C., Spakowitz A.J., Theriot J.A. (2010). Bacterial chromosomal loci move subdiffusively through a viscoelastic cytoplasm. Phys. Rev. Lett..

[10] Burnecki K., Kepten E., Janczura J., Bronshtein I., Garini Y., Weron A. (2012). Universal algorithm for identification of fractional Brownian motion. A case of telomere subdiffusion. Biophys. J..

[11] Hajjoul H., Mathon J., Ranchon H., Goiffon I., Mozziconacci J., Albert B., Carrivain P., Victor J.M., Gadal O., Bystricky K. (2013). High-throughput chromatin motion tracking in living yeast reveals the flexibility of the fiber throughout the genome. Genome Res..

[12] Chen B., Gilbert L.A., Cimini B.A., Schnitzbauer J., Zhang W., Li G.W., Park J., Blackburn E.H., Weissman J.S., Qi L.S. (2013). Dynamic imaging of genomic loci in living human cells by an optimized CRISPR/Cas system. Cell.

[13] Lucas J.S., Zhang Y., Dudko O.K., Murre C. (2014). 3D trajectories adopted by coding and regulatory DNA elements: First-passage times for genomic interactions. Cell.

[14] Backlund M.P., Joyner R., Moerner W.E. (2015). Chromosomal locus tracking with proper accounting of static and dynamic errors. Phys. Rev..

[15] De Gennes P.G. (1979). Scaling Concepts in Polymer Physics.

[16] Girst S., Hable V., Drexler G.A., Greubel C., Siebenwirth C., Haum M., Friedl A.A., Dollinger G. (2013). Subdiffusion Supports Joining Of Correct Ends During Repair Of DNA Double-Strand Breaks. Sci. Rep..

[17] Di Pierro M., Potoyan D.A., Wolynes P.G., Onuchic J.N. (2018). Anomalous diffusion, spatial coherence, and viscoelasticity from the energy landscape of human chromosomes. Proc. Natl. Acad. Sci. USA.

[18] Waigh T.A., Korabel N. (2023). Heterogeneous anomalous transport in cellular and molecular biology. Rep. Prog. Phys..

[19] Amitai A., Seeber A., Gasser S.M., Holcman D. (2017). Visualization of chromatin decompaction and break site extrusion as predicted by statistical polymer modeling of single-locus trajectories. Cell Rep..

[20] Caridi C.P., Plessner M., Grosse R., Chiolo I. (2019). Nuclear actin filaments in DNA repair dynamics. Nat. Cell Biol..

[21] Shinkai S., Nozaki T., Maeshima K., Togashi Y. (2016). Dynamic nucleosome movement provides structural information of topological chromatin domains in living human cells. Plos Comput. Biol..

[22] Hihara S., Pack C.G., Kaizu K., Tani T., Hanafusa T., Nozaki T., Takemoto S., Yoshimi T., Yokota H., Imamoto N. (2012). Local nucleosome dynamics facilitate chromatin accessibility in living mammalian cells. Cell Rep..

[23] Javer A., Kuwada N.J., Long Z., Benza V.G., Dorfman K.D., Wiggins P.A., Cicuta P., Lagomarsino M.C. (2014). Persistent super-diffusive motion of Escherichia coli chromosomal loci. Nat. Commun..

[24] Heun P., Laroche T., Shimada K., Furrer P., Gasser S.M. (2001). Chromosome dynamics in the yeast interphase nucleus. Science.

[25] Levi V., Ruan Q., Gratton E. (2005). 3-D particle tracking in a two-photon microscope: Application to the study of molecular dynamics in cells. Biophys. J..

[26] Zidovska A., Weitz D.A., Mitchison T.J. (2013). Micron-scale coherence in interphase chromatin dynamics. Proc. Natl. Acad. Sci. USA.

[27] Shi G., Liu L., Hyeon C., Thirumalai D. (2018). Interphase human chromosome exhibits out of equilibrium glassy dynamics. Nat. Commun..

[28] Cho N.W., Dilley R.L., Lampson M.A., Greenberg R.A. (2014). Interchromosomal homology searches drive directional ALT telomere movement and synapsis. Cell.

[29] Oshidari R., Strecker J., Chung D.K., Abraham K.J., Chan J.N., Damaren C.J., Mekhail K. (2018). Nuclear microtubule filaments mediate non-linear directional motion of chromatin and promote DNA repair. Nat. Commun..

[30] Seeber A., Hauer M.H., Gasser S.M. (2018). Chromosome dynamics in response to DNA damage. Annu. Rev. Genet..

[31] Soutoglou E., Misteli T. (2007). Mobility and immobility of chromatin in transcription and genome stability. Curr. Opin. Genet. Dev..

[32] Aten J.A., Stap J., Krawczyk P.M., van Oven C.H., Hoebe R.A., Essers J., Kanaar R. (2004). Dynamics of DNA double-strand breaks revealed by clustering of damaged chromosome domains. Science.

[33] Aymard F., Aguirrebengoa M., Guillou E., Javierre B.M., Bugler B., Arnould C., Rocher V., Iacovoni J.S., Biernacka A., Skrzypczak M. (2017). Genome-wide mapping of long-range contacts unveils clustering of DNA double-strand breaks at damaged active genes. Nat. Struct. Mol. Biol..

[34] Friedland W., Jacob P., Kundrat P. (2011). Mechanistic simulation of radiation damage to DNA and its repair: On the track towards systems radiation biology modelling. Radiat. Prot. Dosim..

[35] Warmenhoven J.W., Henthorn N.T., Ingram S.P., Chadwick A.L., Sotiropoulos M., Korabel N., Fedotov S., Mackay R.I., Kirkby K.J., Merchant M.J. (2020). Insights into the non-homologous end joining pathway and double strand break end mobility provided by mechanistic in silico modelling. Dna Repair.

[36] Kroese D.P., Botev Z.I. (2014). Spatial process simulation. Stochastic Geometry, Spatial Statistics and Random Fields: Models and Algorithms.

[37] Henthorn N., Warmenhoven J., Sotiropoulos M., Aitkenhead A.H., Smith E., Ingram S., Kirkby N., Chadwick A., Burnet N.G., Mackay R.I. (2019). Clinically relevant nanodosimetric simulation of DNA damage complexity from photons and protons. Rsc Adv..

[38] Toussaint D., Wilczek F. (1983). Particle–antiparticle annihilation in diffusive motion. J. Chem. Phys..

[39] Yuste S.B., Lindenberg K. (2002). Subdiffusion-limited reactions. Chem. Phys..

[40] Chaudhary P., Marshall T.I., Currell F.J., Kacperek A., Schettino G., Prise K.M. (2016). Variations in the processing of DNA double-strand breaks along 60-MeV therapeutic proton beams. Int. J. Radiat. Oncol. Biol. Phys..

[41] Carter R.J., Nickson C.M., Thompson J.M., Kacperek A., Hill M.A., Parsons J.L. (2018). Complex DNA damage induced by high linear energy transfer alpha-particles and protons triggers a specific cellular DNA damage response. Int. J. Radiat. Oncol. Biol. Phys..

[42] Bettega D., Dubini S., Fuhrman Conti A., Pelucchi T., Tallone Lombardi L. (1981). Chromosome aberrations induced by protons up to 31 MeV in cultured human cells. Radiat. Environ. Biophys..

[43] Schmid E., Ross H., Rimpl G., Bauchinger M. (1997). Chromosome aberration frequencies in human lymphocytes irradiated in a multi-layer array by protons with different LET. Int. J. Radiat. Biol..

